# Effects of Pathogenic *Escherichia coli* Infection on the Flora Composition, Function, and Content of Short-Chain Fatty Acids in Calf Feces

**DOI:** 10.3390/ani12080959

**Published:** 2022-04-08

**Authors:** Lina He, Chunjie Wang, Huasai Simujide, Han Aricha, Jian Zhang, Bo Liu, Chen Aorigele

**Affiliations:** 1 College of Animal Science, Inner Mongolia Agricultural University, Hohhot 010018, China; na15374712742@163.com (L.H.); smjd_2010@163.com (H.S.); 15248146527@163.com (H.A.); zhangjian280613@163.com (J.Z.); lb15754882650@163.com (B.L.); 2 College of Veterinary Medicine, Inner Mongolia Agricultural University, Hohhot 010018, China; chunjiewang200@sohu.com

**Keywords:** pathogenic *Escherichia coli* O1, calf, diarrhea, intestinal florae, short-chain fatty acids

## Abstract

**Simple Summary:**

Calf diarrhea caused by bacteria usually begins to occur 2–3 d after birth. The main pathogenic bacteria are *Escherichia coli*, *Campylobacter*, *Salmonella*, and *Clostridium perfringens*. Pathogenic *E. coli* is the main bacterium that causes diarrhea in calves. It invades the body of calves and releases enteric toxins that cause diarrhea. The incidence of calf diarrhea caused by pathogenic *E. coli* O1 can reach 60–70%, even as high as 90–100% in large-scale calf farms, and the mortality rate can reach over 50%. Therefore, the prevention and treatment of calf diarrhea are extremely important. We established a calf diarrhea model by artificially intervening in the colonization of intestinal microbial flora and studied the influence of pathogenic *E. coli* on the composition, function, and short-chain fatty acid content of calf feces. Our research shows that pathogenic *E. coli* O1 can significantly reduce the abundance and diversity of calf intestinal microflora, increase harmful bacteria, reduce the colonization of beneficial bacteria, and reduce the short-chain fatty acid content in feces.

**Abstract:**

Calf diarrhea caused by pathogenic *Escherichia coli* is a major cause of death in calves, with a mortality rate of over 50%. It is crucial to understand the pathogenesis and development of calf diarrhea for its prevention and treatment. We aimed to study the effect of pathogenic *E. coli* on the flora composition, function, and short-chain fatty acid (SCFA) content of calf feces using a calf diarrhea model. Sixty-four newborn Holstein calves (40–43 kg) were divided into a normal group (NG; *n* = 32) and a test group (TG; *n* = 32). At the beginning of the experiment, the TG were orally administered pathogenic *E. coli* O1 (2.5 × 10^11^ CFU/mL, 100 mL) to establish a calf diarrhea model, and the NG were orally administered the same amount of physiological saline solution. The calves of the two groups were subjected to the same feeding and management. Fresh feces samples were collected at different time points and subjected to 16S rRNA high-throughput sequencing and gas chromatography–mass spectrometry to determine the fecal microbial composition and SCFA content. Pathogenic *E. coli* O1 significantly altered microbiotas composition in the feces of calves, increasing the relative abundance of Proteobacteria and decreasing that of Firmicutes. It also led to a significant increase in the relative abundance of *Escherichia-Shigella* and a decrease in *Lactobacillus*, as well as significantly decreased SCFA content. Therefore, we postulate that pathogenic *E. coli* induces calf diarrhea by causing intestinal florae imbalance and reducing the content of SCFA.

## 1. Introduction

Newborn calf diarrhea is the main cause of the death of calves before weaning [1,2]. Diarrhea in calves can cause poor growth and development, reduced feed utilization rate, and increased mortality, which brings huge economic losses to the cattle industry [3]. The pathogenic bacteria that induce diarrhea in calves include *Escherichia coli, Campylobacter, Salmonella,* and *Clostridium perfringens*. Of these, pathogenic *E. coli* is the main bacteria that causes diarrhea in calves by releasing enterotoxins [4].

Newborn calves have an incompletely developed gut that harbors few microbial species, so their immunity is weak, and the intestinal tract is susceptible to colonization by pathogenic bacteria that cause inflammation [5]. The health of animals is closely related to their gastrointestinal ecosystem. Healthy animals are able maintain the ecological balance of microorganisms in the gastrointestinal tract to resist foreign pathogenic invasion [6]. Pathological changes in animals can cause intestinal microecological disorders, and the changes in intestinal florae are more obvious [7,8]. Some studies have shown that changes in intestinal microbes are related to digestive tract diseases, and intestinal diseases alter the balance between the host and the intestinal microbes [9]. However, little is known about whether the changes in calf intestinal microflorae cause diarrhea, and the underlying mechanism of this phenomenon.

Intestinal microorganisms produce short-chain fatty acids (SCFAs) through fermentation, which mainly include acetic acid, propionic acid, butyric acid, and valeric acid [10]. For example, *Lactobacillus* and *Bifidobacterium* produce SCFAs by fermenting dietary fiber, resistant starch, and oligosaccharides [11]. The intestinal florae of ruminants, such as Bacteroides and Prevost in yak intestine, can produce SCFAs by metabolizing carbohydrates in the intestine [12], and the hindgut of Small-tailed Han sheep is also rich in florae that can produce SCFAs [13]. SCFAs produced by intestinal florae participate in host metabolic pathways as signal molecules, regulating host energy metabolism, immunity, and intestinal development [14,15]. SCFAs have anti-inflammatory effects, and SCFA-producing bacteria may reverse the imbalance of intestinal microbial florae and inhibit the secretion of pro-inflammatory cytokines [16]. Therefore, SCFA-producing bacteria may be expected to become a new generation of probiotics for treating gastrointestinal inflammatory diseases and inhibiting pathogenic bacteria.

Therefore, we aimed to assess whether the early infection of calves with pathogenic *E. coli* affects the intestinal florae and SCFA content in calves. Assuming that diarrhea induced by early infection with pathogenic *E. coli* reduces the abundance of beneficial intestinal bacteria and the content of SCFAs, we investigated the interaction between the signature intestinal microbes and their metabolites in calf diarrhea. The study of the influence of pathogenic *E. coli* on the composition and function of calf intestinal florae and the content of SCFAs in feces may provide theoretical reference for the prevention and treatment of calf diarrhea.

## 2. Materials and Methods

### 2.1. Ethics Statement

The calf test was evaluated and approved by the Animal Care and Use Committee of the Inner Mongolia Agricultural University (Hohhot, China). The experimental program was conducted in strict accordance with the National Standard Guidelines for the Ethical Review of Animal Welfare (GB/T 35892-2018).

### 2.2. Test Strain and Preparation

Pathogenic *E. coli* O1 were inoculated on solid nutrient agar medium and stored in the laboratory at 37 °C for 24 h. A single round and plump colony was selected, inoculated in nutrient broth, and incubated at 37 °C for 24 h on a shaker. In the logarithmic phase of the colony growth, a microplate reader was used to measure the density of the bacteria at 600 nm optical density (OD) to determine the required concentration, and the bacteria were stored in a suspension at 4 °C in a refrigerator. Then, eosin methylene blue agar medium was used to detect the strain [17].

### 2.3. Grouping of Test Animals and Sample Collection

Sixty-four newborn Holstein male calves (age: 3–4 days old; weight: 40–43 kg) were randomly divided into a normal group (NG) and a test group (TG), with 32 calves in each group. Calves were raised outside in individual hutches. At the beginning of the experiment, the calves of the TG were orally administered pathogenic *E. coli* O1 (2.5 × 10^11^ colony-forming unit (CFU)/mL, 100 mL) to establish a calf diarrhea model, and the NG was orally administered the same amount of physiological saline solution. The two groups were subjected to the same feeding and management; all calves were fed twice a day with milk substitute (Eastern Bell, Beijing, China), 3 L each time, and were allowed to freely drink water and eat concentrates (Hohhot, Inner Mongolia, China). The experimental period was 30 d.

At 12 h, 24 h, 36 h, 48 h, 72 h, 5 d, 10 d, and 30 d of the experiment, we collected feces samples immediately after the calves’ natural defecation, placed them in a freezing tube, put them into a liquid nitrogen tank, and brought them back to the laboratory for storage at −80 °C.

### 2.4. DNA Extraction and High-Throughput Sequencing Analysis of Feces

Fecal DNA was extracted according to the operation steps of the E.Z.N.A.^®^ soil DNA kit (Omega Bio-Tek, Norcross, GA, USA). Polymerase chain reaction (PCR) was performed according to the method of Wu et al. using the extracted DNA as a template; the primers used to carry out the PCR amplification of the V3–V4 region of the bacterial 16S rRNA gene were as follows: F, 5′-ACTCCTACGGGAGGCAGCAG-3′ and R, 5′-GGACTACHVGGGTWTCTAAT-3′ [18]. The Illumina MiSeq PE300 platform/NovaSeq PE250 platform (Illumina, San Diego, CA, USA) was used for sequencing according to the standard protocol of Meggie Biological Co., Ltd. (Shanghai, China) [19].

According to the Quantitative Insights into Microbial Ecology (QIIME) quality control process, the data obtained from sequencing were filtered [20,21]. The effective sequences were clustered into operational classification units (OTU) using Uparse software (Uparse V7.0.1090) according to the similarity of ≥97% [22]. The default parameters of QIIME (Version 1.7.0) were used to calculate the Alpha Diversity Index, including Ace and Chao analysis of the flora abundance index, Shannon and Simpson analysis of the flora diversity index, and good coverage analysis of the coverage index [22], as well as the species distribution of each sample. Based on the classification information, we performed a statistical analysis of community structure (heat map, bar chart) and linear discriminant analysis effect (LEfSe) for the different classification levels [23]. Sequencing service, database construction, and statistical analyses were carried out by Shanghai Maggie Biomedical Technology Co., Ltd. (Shanghai, China).

### 2.5. PICRUSt and Correlation Analysis Based on 16S Amplicon Sequencing Results

PICRUSt is a software package for predicting the functional composition of microbial communities in samples from 16S amplicon sequencing results [24]. First, the OTU abundance table was standardized by PICRUSt to remove the influence of the number of copies of the 16S marker gene in the species genome. Then, the Cluster of Orthologous Groups (COG) family information and KEGG Ortholog (KO) information corresponding to each OTU were obtained through the corresponding Greengene ID of each OUT, and the abundance of each COG and KO was calculated. According to the information of the COG database, the description of each COG and its function information was analyzed from the eggNOG database and the function abundance spectrum was obtained. Based on the information of the Kyoto Encyclopedia of Genes and Genomes (KEGG) database, KO and Pathway information were obtained and the abundance of each functional category according to the abundance of OTU was calculated.

Correlation Heatmap analysis and the software R (version 3.3.1) were used to calculate the correlation coefficient between environmental factors and the selected species (Spearman rank correlation coefficient, Pearson correlation coefficient and the obtained numerical matrix was visually displayed through the Heatmap chart. The color changes reflect data information in a two-dimensional matrix or table, and color intensity indicates the size of data values, intuitively expressed by defined color shades.

All the above analyses were performed using software in the cloud platform of Shanghai Maggie Biomedical Technology Co., Ltd. (Shanghai, China).

### 2.6. Determination of SCFAs in the Rectal Feces by Gas Chromatography–Mass Spectrometry (GC-MS)

For the sample pretreatment, the rectal feces sample (0.5 g) was placed in a 10 mL centrifuge tube, and 8 mL of acid diluent (acid diluent: 15 mL of a mixed solution of 100 mmol/L 2-ethyl butyric acid and 50 mL of 5 mmol/L hydrochloric acid) was added to the centrifuge tube, and the tube was mixed evenly on a vortex shaker for 3 min, so that the fecal sample and the acid diluent were fully mixed to prepare the fecal suspension. Then, the sample was centrifuged at 4 °C for 20 min and 15,000 r/min. After centrifugation, 1 mL of the supernatant was collected, filtered through a 0.22 µm microporous filter membrane of the organic phase, and placed in the injection bottle for inspection.

For the pretreatment of the standard substance, the SCFAs were determined by the internal standard method, and the standard substances, that is, 330 µL of acetic acid, 400 µL of propionic acid and 160 µL of butyric acid were added into a 100 mL volumetric flask, which was mixed evenly, and the mixed standard stock solution was prepared in a constant volume with ultrapure water. Then, 0.2, 0.15, 0.1, 0.05, and 0.025 mL of mixed standard stock solution were added into centrifuge tubes, respectively. Then, 0, 0.05, 0.1, 0.15, and 0.175 mL of ultrapure water were added into the corresponding centrifugal tubes to prepare five standard solutions with different gradients. With 2-ethyl butyric acid as the internal standard, 200 µL was added into the standard solutions with different concentrations, mixed evenly, and analyzed with a gas-intake mass spectrometer. The program design for the determination of SCFAs by GC-MS is shown in Table 1.

The instrument automatically detects and analyzes the sample data and finally obtains the corresponding mass spectrum of the sample information. By comparing the obtained mass spectrogram with the standard database, the substances represented by each peak in the mass spectrogram can be determined, and the content of each substance can be calculated according to the drawn standard curve.

### 2.7. Statistical Analysis

Microbiological data analysis, PICRUSt and correlation analysis were performed using the software in the cloud platform of Shanghai Maggie Biomedical Technology Co., Ltd. (Shanghai, China). Excel 2019 software was used for the preliminarily processing of the experimental data for SCFA determination, and SPSS software (version 20.0) was used for the one-way analysis of variance (ANOVA). GraphPad Prism 9 was used to generate statistical graphs. The test results were expressed as mean ± standard deviation, wherein *p* < 0.05 and *p* > 0.05 indicated that the difference was significant, and not significant, respectively.

## 3. Results

### 3.1. Effect of Pathogenic E. coli O1 on Calf Rectal Microflorae

Table 2 shows the alpha diversity analysis of the calf rectal fecal microbes in the NG and TG under the 97% consistency threshold. The ACE index, Chao index, Shannon index, and Simpson index in the TG were significantly different from those in the NG at the first 36 h and 30th day (*p* < 0.05), but not at other times (*p* > 0.05). The ACE and Chao indices of TG were lower than those of NG, which indicated that the number of microbes in the calf feces of TG was lower than that of NG. The Shannon index value of the TG was lower than that of the normal group, while the Simpson index value in the TG was higher than that in the NG, which indicated that the microbial diversity of calf feces in the TG was lower than that of the NG. The ACE and Chao indices are proportional to microbial richness, and the Shannon and Simpson indices indicate microbial diversity. Community diversity increases with an increase in the Shannon index and decreases with an increase in the Simpson index. In this study, the ACE index, Chao index, and Shannon index increased with calf age, while the Simpson index showed the opposite trend. These results indicate that the microbial diversity of calves increased with age, but diarrhea induced by pathogenic *E. coli* O1 reduced the richness and diversity of the rectal microbial flora of the calves.

**Figure 1:**
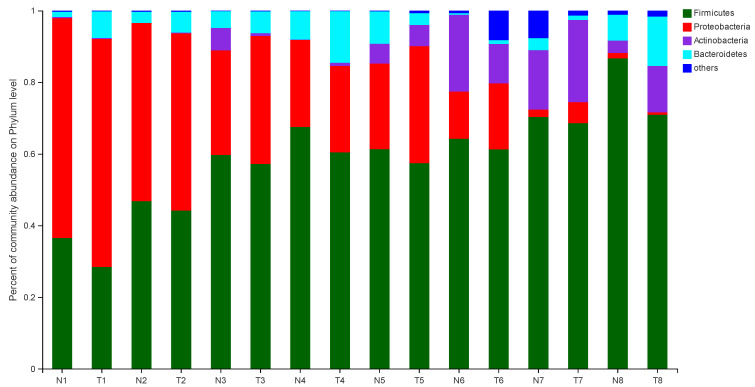
Abundance of the different phyla (%) in fecal florae. N: Normal group (NG); T: Test group (TG). Numbers 1–8 represent the sampling times of 12 h, 24 h, 36 h, 48 h, 72 h, 5 d, 10 d, and 30 d, respectively. Time relative to start of the study.

**Figure 2:**
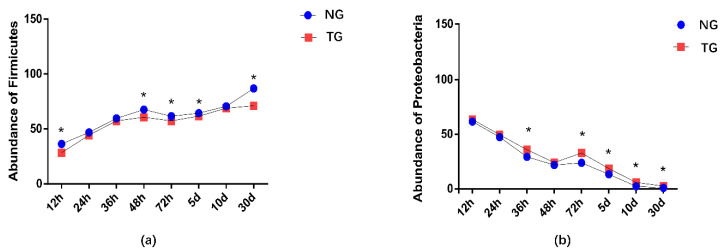
Abundance of the different phyla (%) in calf rectal flora. ‘*’ indicates a significant difference. NG: Normal group; TG: Test group; 12 h, 24 h, 36 h, 48 h, 72 h, 5 d, 10 d, and 30 d, represent the sampling times, respectively. Time relative to start of the study.

The effect of pathogenic *E. coli* O1 on the microflorae of calf rectal feces is shown in Figure 1 and Figure 2. Firmicutes, Proteobacteria, Actinobacteria, and Bacteroidetes were the dominant phyla in the TG and NG, while the rest had an abundance of less than 1%. Among these, there were three phyla with obvious differences. Compared with that in the NG, the abundance of Firmicutes and Bacteroidetes in the TG were significantly reduced (*p* < 0.05), and the abundance of Proteobacteria was significantly increased (*p* < 0.05). The relative abundance of Firmicutes gradually increased with calf age, while that of Proteobacteria gradually decreased with calf age.

**Figure 3:**
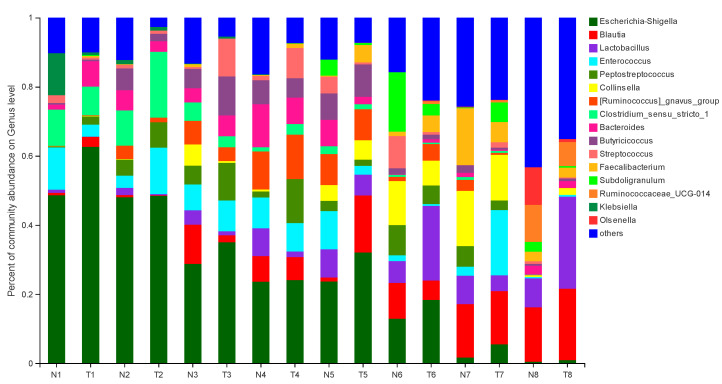
Abundance of different genera (%) in calf rectal flora. N: Normal group (NG); T: Test group (TG). Numbers 1–8 represent the sampling times of 12 h, 24 h, 36 h, 48 h, 72 h, 5 d, 10 d, and 30 d, respectively. Time relative to start of the study.

**Figure 4:**
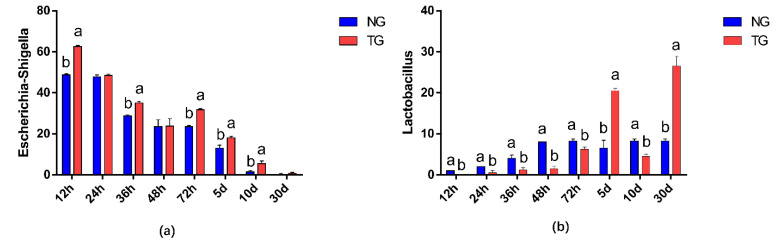
Abundance of different genera (%) in calf rectal flora. The different letters indicate significant differences. NG: Normal group; TG: Test group; 12 h, 24 h, 36 h, 48 h, 72 h, 5 d, 10 d, and 30 d, represent the sampling times, respectively. Time relative to start of the study. Error bars indicate SD.

The effect of pathogenic *E. coli* O1 on the abundance of different genera in calf rectal feces is shown in Figure 3 and Figure 4. The top 10 dominant genera with relative abundance in the TG and NG were *Escherichia*-*Shigella*, *Blautia*, *Lactobacillus*, *Enterococcus*, *Peptostreptococcus*, *Collinsella*, *Ruminococcus-gnavus*-group, *Clostridium sensu stricto* 1, *Bacteroides*, and *Butyricicoccus*. Among them, there were two types of bacteria with obvious differences. Compared with that in the NG, TG *Escherichia*-*Shigella* significantly increased (*p* < 0.05) and *Lactobacillus* was significantly decreased in the early stage and significantly increased in the later stage (*p* < 0.05). The relative abundance of *Escherichia*-*Shigella* decreased gradually with calf age, while that of *Lactobacillus* increased gradually with calf age.

**Figure 5:**
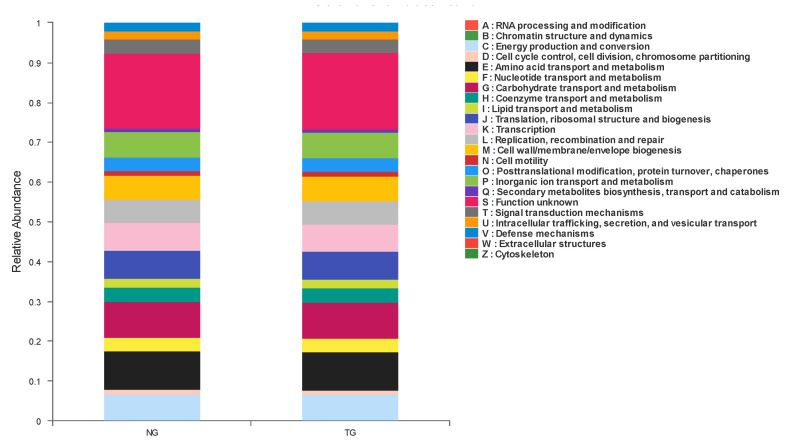
Cluster of Orthologous Groups (COG) function classification column diagram.

According to the functional classification of proteins predicted by COG, 23 groups were obtained, as shown in Figure 5. In these COG classifications, the groups with the highest abundance in calf feces from the TG and NG mainly included amino acid transport and metabolism, carbohydrate transport and metabolism, transcription, translation, ribosomal structure and biogenesis, and energy production and conversion, among others. The only statistically significant factor in COG prediction function abundance between the NG and TG was transcription, which decreased after calf diarrhea was induced by pathogenic *E. coli*.

**Figure 6:**
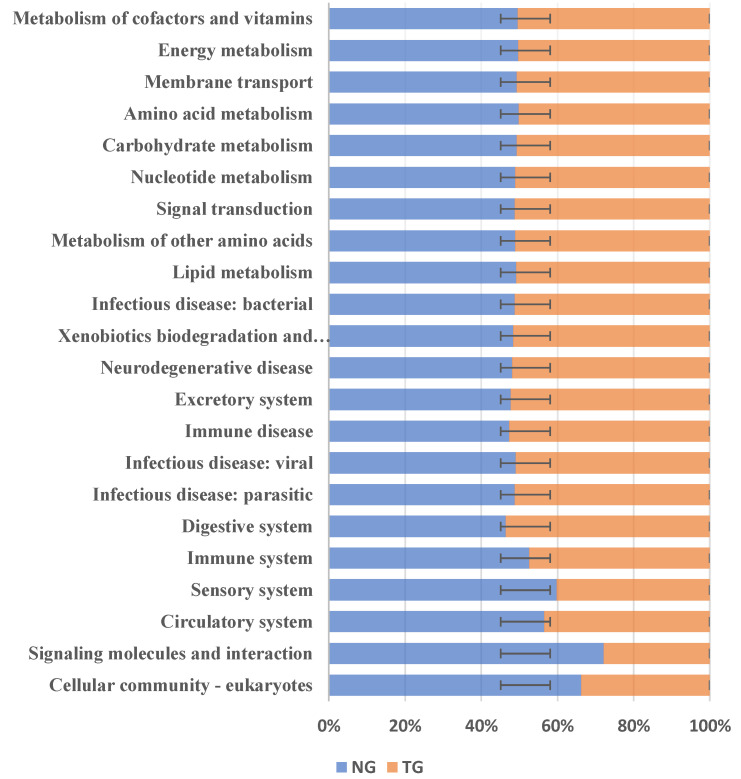
Kyoto Encyclopedia of Genes and Genomes (KEGG) metabolic pathway analysis. NG: Normal group; TG: Test group. The error bars indicate positive and negative deviations.

The phylogenetic investigation of communities by the reconstruction of unobserved states (PICRUSt) can predict the relative abundance of microbial functional categories based on the Kyoto Encyclopedia of Genes and Genomes (KEGG) database, according to 16S rDNA sequencing data. We used PICRUSt to predict the function of the intestinal microbes of the TG and the NG, and compared these predictions with the KEGG database to obtain a total of 22 differential metabolic pathways. The results are shown in Figure 6. Compared with the NG, KEGG analysis of the TG showed that five metabolic pathways were decreased: cell community-eukaryotes, signaling molecules and interactions, the circulatory system, the sensory system, and the immune system. On the other hand, there were 17 metabolic pathways that were increased in the TG: digestive system infectious disease: parasites; infectious diseases: viral diseases, immune disease, excretory system, neurodegenerative disease, xenobiotic biodegradation and metabolism; infectious diseases: bacterial, lipid metabolism, metabolism of other amino acids, signal transduction, nucleotide metabolism, carbohydrate metabolism, amino acid metabolism, membrane transport, energy metabolism, and metabolism of cofactors and vitamins. The results showed that after pathogenic *E. coli* infection, the abundance of metabolic pathways in the immune system decreased, and the abundance of pathways related to carbohydrate metabolism, exogenous substance degradation, and metabolism and energy metabolism increased.

### 3.2. Effect of Pathogenic E. coli O1 on the Content of SCFAs in Calf Rectal Feces

**Figure 7:**
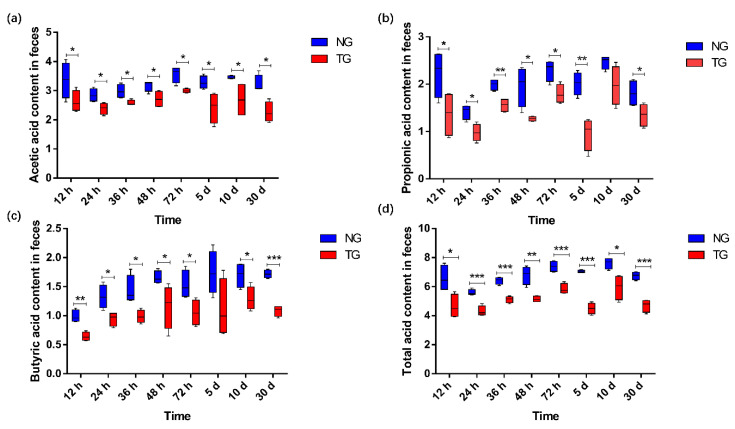
Comparison column chart of SCFA differences: (**a**) acetic acid; (**b**) propionic acid; (**c**) butyric acid; (**d**) total acid. The units for the measures of the SCFAs are mmol/kg. * 0.01< *p* ≤ 0.05, ** 0.001 < *p* ≤ 0.01, *** *p* ≤ 0.001. NG: Normal group; TG: Test group; 12 h, 24 h, 36 h, 48 h, 72 h, 5 d, 10 d, and 30 d represent the sampling times, respectively. Time relative to start of the study.

The effect of pathogenic *E. coli* on the SCFA content in the calf rectal feces is shown in Figure 7. Compared with that in the NG, the content of acetic acid and total acid (*p* < 0.05), propionic acid (except for that at 10 d) (*p* < 0.05), and butyric acid (except for that at 5 d) (*p* < 0.05) in the TG were significantly reduced.

### 3.3. Correlation Analysis between Calf Rectal Microflora and SCFA Content in Feces

**Figure 8:**
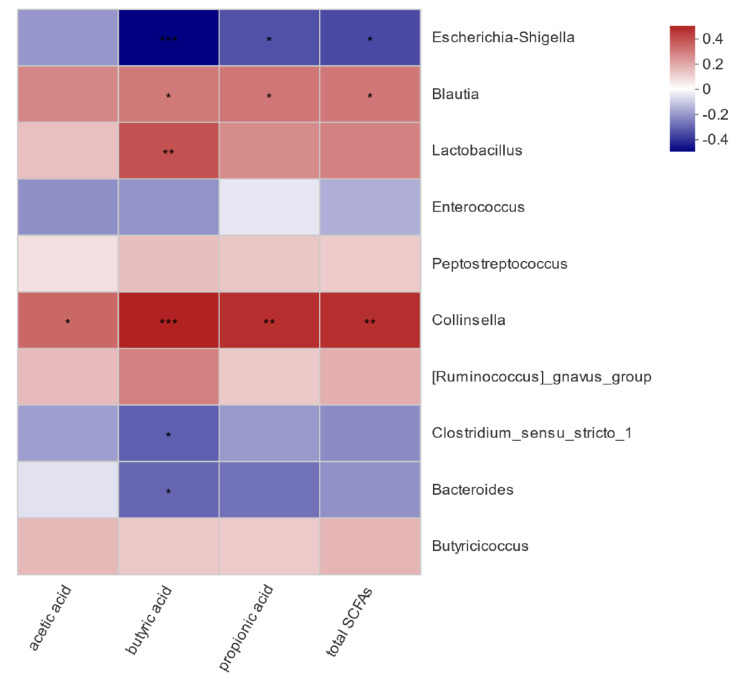
Heat map of correlation analysis between rectal microorganisms and short-chain fatty acids. * 0.01 < *p* ≤ 0.05, ** 0.001 < *p* ≤ 0.01, *** *p* ≤ 0.001.

To assess if the influence of pathogenic *E. coli* O1 on calf rectal microbes is related to the SCFA content in the rectal feces, a correlation analysis between bacteria with significant differences in abundance and SCFA content in the different groups was carried out, and the results are shown in Figure 8. Acetic acid content was significantly and positively correlated with *Collinsella* (*p* < 0.05). Propionic acid content was positively correlated with *Collinsella* and *Blautia* (*p* < 0.05) and negatively correlated with *Escherichia*-*Shigella* (*p* < 0.05). Butyric acid content was significantly positively correlated with *Collinsella*, *Lactobacillus*, and *Blautia* (*p* < 0.05), significantly negatively correlated with *Escherichia*-*Shigella* (*p* < 0.001), and significantly negatively correlated with *Bacteroides* and *Clostridium sensu stricto 1* (*p* < 0.05). Total acid content was positively correlated with *Collinsella* and *Blautia* (*p* < 0.05) and negatively correlated with *Escherichia*-*Shigella* (*p* < 0.05).

## 4. Discussion

The intestinal tract is the largest immune organ in the body and is the main site for the digestion and absorption of nutrients. The health of the intestine largely determines the growth performance and overall health of animals. There are many microorganisms in the gastrointestinal tract of animals, and an animal’s health is closely related to the state of their gastrointestinal ecosystem. A healthy animal’s body maintains the ecological balance of microorganisms in the gastrointestinal tract to resist foreign pathogenic invasion [25]. Diseases can cause intestinal microecological disorders, especially when diarrhea occurs, and the changes in intestinal florae are more obvious [7,8]. This study showed that the microbial diversity in the feces of diarrheal calves in the TG was lower than that of healthy calves in the NG, which is consistent with the results of Han et al. [26] in yaks, wherein the microbial diversity in the feces of healthy peripartum yaks was higher than that of diarrheal peripartum yaks. High diversity and stability of the intestinal microbial community indicate that calves are healthy [27]. It is speculated that diarrhea in calves induced by *E. coli* may be an important factor leading to intestinal microbial imbalance.

The two dominant phyla in the gastrointestinal tract of mammals are Firmicutes and Bacteroides [28]. In our study, the main phyla in the NG and TG gut mainly included Firmicutes, Proteobacteria, Actinomycetes, and Bacteroidetes, of which Firmicutes and Proteobacteria were dominant. Firmicutes are mainly involved in carbohydrate fermentation and protein utilization processes [29,30]. A gut microbiome rich in Firmicutes allows cattle to absorb more energy from the diet, and thus maintain better bodily functions [22]. Our study found that the relative abundance of Firmicutes in TG was lower than that in NG at the same time point, and the abundance of Firmicutes gradually increased with the age of calves. This indicates that calf diarrhea caused by pathogenic *E. coli* infection affects the digestion and absorption of nutrients in the body, but as calves grow, the body’s demand for nutrients and energy increases, and the abundance of Firmicutes increases. Shin et al. found that Proteobacteria is the main bacteria in intestinal microflorae, which is closely related to enteritis, immune imbalance and florae imbalance [31]. The lower the abundance of Proteobacteria, the healthier the intestinal tract; therefore, Proteobacteria abundance is used as an important index to measure intestinal health [31]. The relative abundance of Proteobacteria in calf feces increased significantly in the TG. Proteobacteria include many pathogens, such as *E. coli*, *Salmonella, Helicobacter pylori* and *Vibrio cholerae* [32]. In this study, the relative abundance of TG Proteobacteria was lower than that of NG at the same time point, and its abundance decreased with calf age, which is consistent with the results of previous research [32]. We postulate that the pathogenic *E.coli* infection caused calf diarrhea, which caused the intestinal inflammation of TG calves, increasing the number of Proteobacteria. With age, the calves’ immunity improved and the number of Proteobacteria decreased.

The bacteria commonly causing calf diarrhea include *E. coli*, *Salmonella*, and *C. perfringens*. Of these, the main pathogenic bacteria causing newborn calf diarrhea are enterotoxigenic *E. coli* [33]. We found that, compared with the fecal microbes of healthy calves in the NG, the abundance of *E. coli* in diarrheal calves in the TG was significantly higher, which indicated that diarrhea in TG calves was caused by *E. coli*, and the model was successfully established. This is consistent with the findings of Kim et al. who showed that *E. coli* increased when calves had diarrhea [34]. *Lactobacillus* functions well in maintaining normal intestinal florae; it inhibits pathogen adhesion to the small intestinal wall, decomposes sugar and prevents inflammation, and mostly lives in the small intestine [35]. Our results showed that TG *Lactobacillus* was significantly lower than NG in the early stages of the experiment, and its abundance increased in the later stages of the experiment, showing that TG was higher than NG. We speculate that *Lactobacillus* may play a protective role in the later stage of calf diarrhea caused by pathogenic *E. coli*, and further research is needed to explore the relationship between calf diarrhea and *Lactobacillus*.

The diarrhea of calves induced by pathogenic *E. coli* not only changes the abundance and composition of intestinal microbes but also affects the function of intestinal microbes. PICRUSt functional analysis of fecal of calves in the TG and NG showed that there were obvious differences in amino acid transport and metabolism, carbohydrate transport and metabolism, and energy generation and conversion functions between the two groups. Through the differential analysis of the KEGG metabolic pathway, the reduced pathways in the TG calves comprised cell community-eukaryotes, signaling molecules and interactions, the circulatory system, the sensory system, and the immune system. The increased pathways in the TG calves comprised immune disease, the excretory system, neurodegenerative disease, xenobiotics biodegradation and metabolism, lipid metabolism, carbohydrate metabolism, amino acid metabolism, membrane transport, energy metabolism, and the metabolism of cofactors and vitamins. This shows that diarrhea leads to an increase in sugar metabolism and lipid metabolism. When calves have diarrhea, they consume more fat to maintain their growth and development, which is consistent with the results of Mei et al. [36].

There is a close relationship between the function of intestinal florae and the host. Besides proteins, intestinal microbes can also synthesize a variety of vitamins, and their metabolites—SCFA salts—also play an important role in the intestinal tract, which is also closely related to immunity [15]. Short-chain fatty acids are one of the main products of microbial metabolism, also known as volatile fatty acids; they mainly include acetic acid, propionic acid, butyric acid, isovaleric acid, valeric acid, and isovaleric acid [10]. Because the rumen of young ruminants is underdeveloped, milk-based feed is mainly digested in the intestine [37]. SCFAs are the final product of microbial fermentation, which provides energy for intestinal cells [38]. The SCFA content in feces can be used as a biomarker of physiological processes and nutritional intervention effects in organisms [38]. Moreover, the SCFA content in feces is related to some diseases such as irritable bowel syndrome (IBS), cardiovascular diseases, diarrhea [39], and cancer [40]. At the same time, previous studies have shown that SCFAs can regulate the host’s biological response, including intestinal integrity, lipid metabolism, and the immune system [41,42,43,44,45]. Butyrate is the main energy source of colon epithelial cells, which can reduce the permeability of intestinal mucosa, promote the recovery of the intestinal barrier, and prevent or reduce the incidence of colon cancer [46]. Metabolites of butyric acid can improve intestinal digestion and the absorption of nutrients in the feed and supplement nutrients needed by the body [47]. Our results showed that pathogenic *E. coli* induced calf diarrhea, and the contents of SCFAs in feces were significantly lower than those of healthy calves in the NG, such as acetic acid, propionic acid, butyric acid, and total acid. This is consistent with the findings of Li et al., who identified that the content of SCFAs in the feces of diarrhea-type yaks was significantly lower than that of healthy yaks [48]. Through correlation analysis of the bacteria with significant differences in the abundance and content of SCFAs, we can further demonstrate that calf diarrhea induced by pathogenic *E. coli* affects intestinal florae—particularly reducing SCFAs and producing bacteria abundance—thereby affecting the production of SCFAs. We found that propionic acid, butyric acid, and total SCFAs were significantly negatively correlated with *E. coli* and *Shigella*. However, *E. coli*-*Shigella* abundance in the TG was significantly higher than that in the NG, and therefore, the content of SCFAs in calf feces in the TG was significantly lower than that in the NG. In summary, our results indicated that calf diarrhea leads to an imbalance of intestinal microbes, a decrease in beneficial bacteria, an increase in harmful bacteria, a decrease in the number of bacteria producing SCFAs, and the impairment of the fermentation pathway of SCFAs. These factors, in turn, lead to a significant decrease in SCFA content in calf feces, compared with that of the healthy calves in the NG.

Although our findings found that calves with diarrhea caused by pathogenic *E. coli* infection had altered fecal microbial community composition and function, and altered metabolic pathways compared with healthy calves, there are still limitations to this study. First, the fecal microbiome is not representative of the entire gut microbiome, so it is necessary for us to conduct comparative research between the fecal and gut microbiome. Moreover, it is necessary to accurately determine the changes in metabolic function through metabonomics to better understand the dynamic interaction between calf *E. coli-*associated diarrhea and intestinal metabolites.

## 5. Conclusions

In conclusion, the early infection of pathogenic *E. coli* in calves, which could induce diarrhea, will reduce the abundance and diversity of intestinal microbes in calves and affect the composition and function of the intestinal microbes. Pathogenic *E. coli* manifests its effect by increasing the relative abundance of Proteobacteria and decreasing that of Firmicutes at the phylum level. At the genus level, the increase in the relative abundance of *Escherichia coli* led to a corresponding significant decrease in the relative abundance of *Lactobacillus* in the early stages of the experiment, and a significant increase in the later stages of the experiment. At the same time, it also reduced the content of SCFAs such as acetic acid, propionic acid, and butyric acid in the feces. The findings of this study lay a theoretical foundation for further research on the molecular mechanism related to pathogenic *E.* *coli*-induced calf diarrhea.

## Figures and Tables

**Table 1:** Program design for the determination of short-chain fatty acids by gas chromatography–mass spectrometry (GC-MS).

Item	Operation
Chromatography system	Agilent 7890B GC
Mass spectrometry system	Agilent 5977B MSD
Chromatographic column	DB-FFAP capillary column, 60 m × 0.25 mm × 0.50 µm
Advance sample port temperature	220 °C
Ion source temperature	230 °C
Temperature rising program	Initial temperature of 80 °C for 3 min, raised from 20 °C to 120 ℃ per minute for 3 min, and then from 4 °C to 180 °C per minute for 3 min
Split ratio	No diversion
Sample size	1.5 µL
Solvent delay	6 min
Run time	26 min

**Table 2:** Analysis of the microbial diversity index of the calf feces (%).

Time	Group	Ace	Chao1	Shannon	Simpson
12 h	NG TG	147.68 ± 10.41 ^a^ 145.42 ± 12.09 ^b^	144.09 ± 9.39 ^a^	1.91 ± 0.11 ^a^	0.31 ± 0.03 ^b^
			167.55 ± 12.92 ^b^	1.51 ± 0.10 ^b^	0.45 ± 0.02 ^a^
24 h	NG TG	153.34 ± 14.72 ^a^ 108.01 ± 2.32 ^b^	140.08 ± 11.56 ^a^	2.00 ± 0.11 ^a^	0.30 ± 0.01 ^a^
			109.33 ± 9.38 ^b^	1.68 ± 0.09 ^b^	0.34 ± 0.03 ^a^
36 h	NG TG	236.24 ± 6.33 ^a^ 179.34 ± 3.22 ^b^	202.80 ± 5.51 ^a^	2.32 ± 0.09 ^a^	0.21 ± 0.03 ^b^
			158.94 ± 12.04 ^b^	2.05 ± 0.10 ^b^	0.25 ± 0.02 ^a^
48 h	NG TG	231.00 ± 11.14 ^a^ 205.68 ± 11.12 ^b^	193.65 ± 9.24 ^a^	2.38 ± 0.15 ^a^	0.18 ± 0.02 ^a^
			201.57 ± 6.75 ^a^	2.23 ± 0.09 ^a^	0.20 ± 0.02 ^a^
72 h	NG	281.59 ± 12.32 ^a^	261.00 ± 11.55 ^a^	2.66 ± 0.16 ^a^	0.14 ± 0.02 ^b^
	TG	241.67 ± 14.97 ^b^	215.41 ± 14.93 ^b^	1.85 ± 0.16 ^b^	0.19 ± 0.01 ^a^
5 d	NG	203.92 ± 17.22 ^a^	197.29 ± 11.69 ^a^	2.07 ± 0.15 ^a^	0.22 ± 0.02 ^a^
	TG	181.13 ± 19.61 ^a^	160.38 ± 10.94 ^b^	2.18 ± 0.08 ^a^	0.24 ± 0.03 ^a^
10 d	NG	237.97 ± 10.38 ^a^	217.32 ± 7.95 ^a^	2.47 ± 0.19 ^a^	0.21 ± 0.01 ^a^
	TG	194.45 ± 9.79 ^b^	182.58 ± 12.48 ^b^	2.45 ± 0.21 ^a^	0.24 ± 0.03 ^a^
30 d	NG	367.79 ± 12.45 ^a^	362.19 ± 8.23 ^a^	3.72 ± 0.19 ^a^	0.06 ± 0.01 ^b^
	TG	331.58 ± 8.82 ^b^	339.42 ± 12.60 ^b^	2.82 ± 0.14 ^b^	0.20 ± 0.02 ^a^

Under each time period and compared with that of the NG, different lowercase letters indicate a significant difference (*p* < 0.05), while the same lowercase letter indicates no significant difference (*p* > 0.05). NG: Normal group; TG: Test group; 12 h, 24 h, 36 h, 48 h, 72 h, 5 d, 10 d, and 30 d represent the sampling times, respectively. Time relative to start of the study.

## Data Availability

The datasets that is applied and/or analyzed throughout the prevailing research are available from the corresponding writer upon fair appeal.
